# The joint profiles of cardiorespiratory fitness and muscle strength on daily physical activity levels in patients with symptomatic peripheral artery disease: A cross-sectional study

**DOI:** 10.1371/journal.pone.0298289

**Published:** 2024-03-27

**Authors:** Paulo Longano, Eduardo Caldas Costa, Renan Massena Costa, Breno Quintella Farah, Nelson Wolosker, Gabriel Grizzo Cucato, Marilia Almeida Correia, Hélcio Kanegusuku, Raphael Mendes Ritti-Dias

**Affiliations:** 1 Graduated Program in Rehabilitation Sciences, Universidade Nove de Julho, São Paulo, Brazil; 2 Department of Physical Education, Federal University of Rio Grande do Norte, Natal, Brazil; 3 Graduated Program in Medicine, Universidade Nove de Julho, São Paulo, Brazil; 4 Department of Physical Education, Rural Federal University of Pernambuco, Recife, Brazil; 5 Hospital Israelita Albert Einstein, São Paulo, Brazil; 6 Department of Sport, Exercise & Rehabilitation, Northumbria University, Newcastle Upon Tyne, United Kingdom; Hamasaki Clinic, JAPAN

## Abstract

**Introduction:**

In peripheral artery disease (PAD) patients, the joint profile of low strength and cardiorespiratory fitness on movement behaviors, specifically physical activity levels and sedentary time, remains unclear.

**Purpose:**

To investigate the joint profiles between cardiorespiratory and neuromuscular fitness and daily physical activity among PAD patients.

**Methods:**

Cross-sectional study in a sample of 155 PAD patients. We measured their physical activity level per week using accelerometers, assessed their muscle strength through a sit-to-stand test and cardiorespiratory fitness through a six-minute walk test. Patients were categorized into three groups: those with high strength and cardiorespiratory fitness (NC, n = 28), those with at least one component classified as low (1C, n = 88), and those with both components classified as low fitness (2C, n = 39).

**Results:**

The patients in the 1C and 2C groups spent less time engaged in low-light and moderate activities compared to the NC group (low-light: NC: 2291 ± 680 minutes/week vs. 1C: 1826 ± 649 minutes/week vs. 2C: 1885 ± 651 minutes/week, p = .005; moderate: NC: 2617 ± 796 minutes/week vs. 1C: 2071 ± 767 minutes/week vs. 2C: 2092 ± 776 minutes/week, p = .005) and the patients in the 2C group spent less time engaged in vigorous activities compared to the NC and 1C groups (NC: 155 ± 148 minutes/week vs. 1C: 110 ± 110 minutes/week vs. 2C: 64 ± 70 minutes/week, p = .003).

**Conclusion:**

PAD patients with low strength and/or cardiorespiratory fitness are more likely to spend less time engaging in low-light and moderate physical activities and patients with low fitness in both components are more likely to spend less time engaging in vigorous physical activity.

## Introduction

Peripheral artery disease (PAD) patients with symptoms of intermittent claudication experience reduced physical activity levels compared to individuals of the same age without PAD [1]. Gerage et al. (2019) observed that only 3.4% of patients achieve the minimum of physical activity levels recommended by general and specific guidelines [2]. Physical activity practice is considered a cornerstone of clinical treatment of these patients [3].

PAD patients usually presents decreased muscle mass and increased fat [4] in the lower limbs, in addition to impaiments in cardiovascular regulation and function, resulting in a decrease in levels of cardiorespiratory fitness and muscle strength in the lower limbs [5–7], which are crucial components for engaging in daily physical activities [8, 9].

Despite the prevalence of low strength and cardiorespiratory fitness in PAD patients, the impact of combining these two components on movement behaviors, specifically physical activity levels and sedentary time, remains unclear. In studies conducted on older adults without PAD, it has been observed that individuals with simultaneous low strength and cardiorespiratory fitness are at a higher risk of developing metabolic syndrome [10], functional limitation [11], and impaired mobility [12] compared to those with low strength or cardiorespiratory fitness, suggesting that this can also occur in PAD patients and, consequently, further negatively impact the daily movement behaviors of these patients.

The objective of this study was to investigate the joint profiles between low strength and cardiorespiratory fitness on objectively-measured physical activity levels and sedentary time in individuals with PAD. Our hypothesis is that the combined presence of low strength and cardiorespiratory fitness would further impair daily movement behaviors in PAD patients.

## Materials and methods

### Eligible participants

Data collection was carried out between September 2015 and October 2019. The human research ethics committee approved the study (CAAE: 4 2379015.3.0000.0071). All patients were informed about the procedures involved in conducting the study and signed the informed consent form. This study was reported according to the STROBE statement. Patients were recruited from the clinic for vascular diseases in the city of São Paulo—Brazil. Patients were included if they were older than 50 years, both genders, had an ankle-brachial index (ABI) ≤0.90 in at least one limb, intermittent claudication symptoms (stage II in PAD), absence of non-compressible vessels, amputated limbs, and/or ulcers, and had valid data of objectively measured movement behaviors by accelerometry, cardiorespiratory fitness and muscle strength. Patients were excluded from the study if they did not present identification of ABI value and did not present valid data on physical activity level, six-minute walk test or sit-to-stand test.

## Procedures

### Clinical data and health history

Using a questionnaire [13–15], clinical and sociodemographic data were obtained, including age, sex, job status, partner status, smoking, concomitant diseases, and medication use. The ABI was measured to confirm the PAD severity [16].

### Objectively measured movement behaviors

Daily physical activity and sedentary time were measured using the GT3X+ accelerometer (Actigraph, United States), as previously described [2]. Each patient was instructed to wear the accelerometer for seven consecutive days, removing it only in case of sleeping, bathing, or if was going to perform any aquatic activity. The device was attached to an elastic belt and attached to the right side of the hip following the manufacturer’s instructions. For data analysis, valid data from patients were considered when they presented a minimum of 10 hours of daily activity recordings in at least four days of use, being at least one weekend day. The data were collected at a frequency of 60Hz and were analyzed using 60s epochs. Periods with consecutive values of zero (with a 2 min spike tolerance) for 60 min or longer were interpreted as “accelerometer not worn” and excluded from the analysis. Step count was measured and the time spent in each intensity of physical activity (light, moderate-vigorous) and sedentary time was estimated based on the cut-off points proposed by Copeland and Esliger [17], already used in a previous study with PAD population [2], considering sedentary as 0–100 counts/min, low-light physical activity as 101–1041 counts/min, and moderate/vigorous as ≥ 1042 counts/min using the vertical axis, and analyzed in min/days, adjusting for the number of days and daily hours that the device was worn.

### Cardiorespiratory fitness

Cardiorespiratory fitness was measured using the six-minute walk test [18, 19]. The test is performed in a 30-meter corridor following the protocol that has been previously described [20]. Patients were instructed to complete as many laps as possible. Total walking distance was defined as the maximum distance achieved by the patient at the end of the test. The predicted percentage of total walking distance was assessed using the Britto et al. [21] equation. Patients were classified as having low cardiorespiratory fitness in case the predicted percentage values were among the values included in the lowest tertile considered all values of this parameter (< 55% of the predicted percentage of the six-minute walk test).

### Strength

Strength was assessed using the sit-to-stand test from the *Short Physical Performance Battery*, as previously described [22]. The test consists of the time that the patient can get up and sit from a chair with the upper limbs across their chest five times, as quickly as possible. The sit-to-stand test data were divided into quartiles according to gender (men and women) and age group (sixty, seventy and eighty years). The second, third and fourth quartile was considered as low neuromuscular fitness.

### Statistical analysis

The normality and homogeneity of variance were analyzed using the Shapiro-Wilks and Levene tests, respectively. Patients were categorized into three groups, according to their cardiorespiratory and strength fitness levels: i) high cardiorespiratory fitness and strength (none fitness component classified as low; NC); ii) low cardiorespiratory fitness or low strength (one fitness component classified as low; 1C); iii) low cardiorespiratory and strength (two fitness components classified as low; 2C). The comparison among the groups regarding the movement behavior outcomes were made using One-way ANOVA test with Bonferroni Post-hoc test and Kruskal-Wallis test (i.e., step count and vigorous physical activity).

The association between the fitness components (NC, 1C and 2C) and the movement behavior outcomes were assessed using multiple linear regressions, adjusted for age, sex, and ABI (Model 1), for Model 1 plus hypertension, diabetes and dyslipidemia (Model 2), and for Mode 2 plus coronary artery disease and heart failure (Model 3). A residual analysis was performed by graphical analysis (histogram). The significance level was set at p<0.05.

## Results

**Fig 1:**
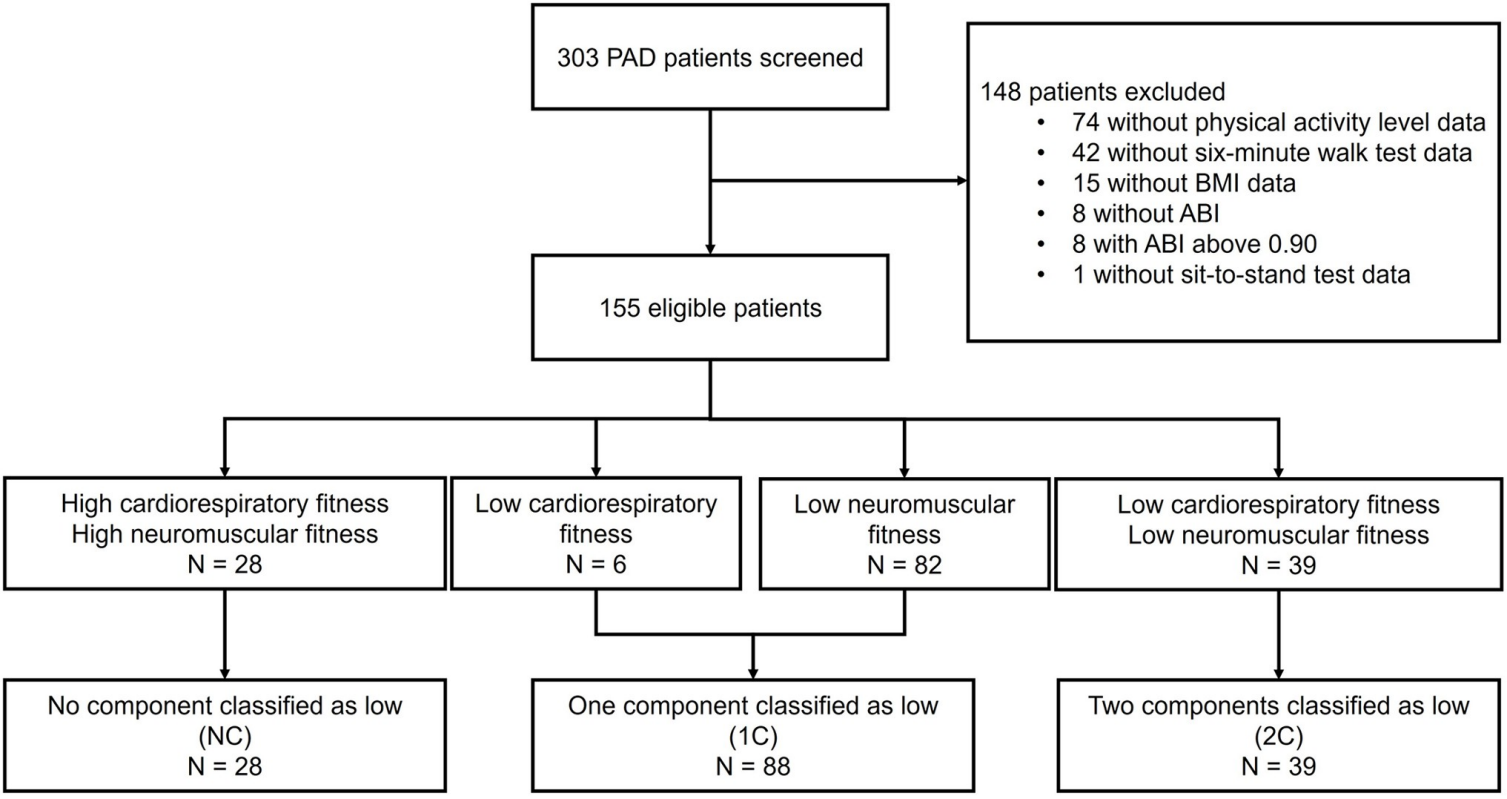
Flowchart of the study.

A total of 303 PAD patients were recruited for this study, and 155 patients were considered eligible for the study and were included in data analysis. The sample distribution among the groups were: 28 patients classified in the NC group, 88 patients in the 1C group, and 39 patients in the 2C group (Fig 1).

The comparisons of the characteristics among the groups are presented in Table 1. Groups were similar regarding the characteristics among the groups (p>0.05). Patients from the NC group had a better performance in the six-minute walk test and sit-to-stand test compared to the patients from the 1C and 2C groups. Patients from the 1C group also had a better performance in both tests than the patients from the 2C group.

**Table 1:** Clinical characteristics of the patients were separated into groups (N = 155).

Variables	NC N = 28	1C N = 88	2C N = 39	*P value*
Sex, men %	68	66	56	.524
Age, yr	65.1 ± 8.6	67.7 ± 8.5	67.1 ± 8.1	.374
Current job, %	29	15	21	.290
With partner, %	50	60	58	.647
Weight, Kg	70.4 ± 12.5	75.2 ± 17.0	71.1 ± 16.2	.241
Height, meters	1.65 ± 0.08	1.62 ± 0.08	1.63 ± 0.10	.218
Body mass index, kg/m^2^	26.3 ± 4.2	28.5 ± 5.0	27.5 ± 4.9	.099
Ankle brachial index	0.58 ± 0.17	0.59 ± 0.16	0.57 ± 0.15	.753
Six-minute walk test, meters	401 ± 49	345± 73*	233 ± 51*^#^	≤.001
Six-minute walk test, %	74 ± 10	65 ± 12*	44 ± 9*^#^	≤.001
Sit-to-stand test, seconds	9.4 ± 2.5	16.7 ± 7.1*	20.6 ± 7.9*^#^	≤.001
***Risk factors (%)***				
Current smoker	21	17	26	.477
Diabetes	32	58	53	.064
Hypertension	86	92	84	.390
Dyslipidemia	93	83	89	.344
Coronary artery disease	36	34	57	.058
Heart Failure	11	16	14	.799
***Medications (%)***				
Antiplatelet	85	92	77	.271
Statins	93	89	83	.536
Vasodilators	19	33	27	.357
Beta-blockers	44	44	40	.926
Calcium blockers	41	33	23	.369
ACEi	22	27	27	.870
ARA	33	36	37	.964
Diuretics	26	47	43	.173
Hypoglycemics	30	51	42	.154

Data presented as mean ± standard deviation or relative frequency; NC—Neither component as low; 1C –one component as low; 2C –both components as low. ACEi—Angiotensin converting enzyme inhibitor; ARA—Angiotensin receptor antagonist.

*Significantly different from NC group.

^#^ Significantly different from the 1C group.

**Fig 2:**
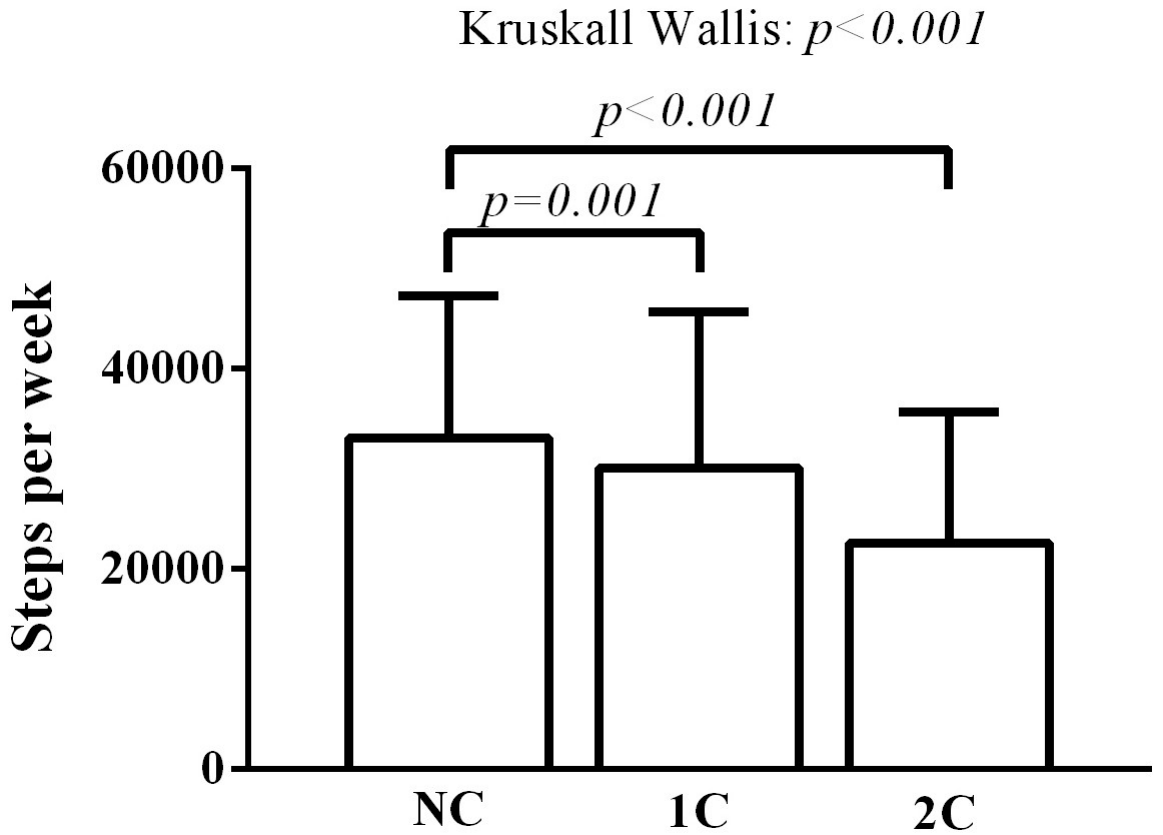
Comparison of daily step count among groups.

Fig 2 shows the comparison of the daily step count among the three groups. Patients from the 1C and 2C groups had a lower step count than patients from the NC group (NC: 39733 ± 15213 steps per week vs. 1C: 28363 ± 14954 steps per week, vs 2C: 23382 ± 12032 steps per week, p =. <001).

**Fig 3:**
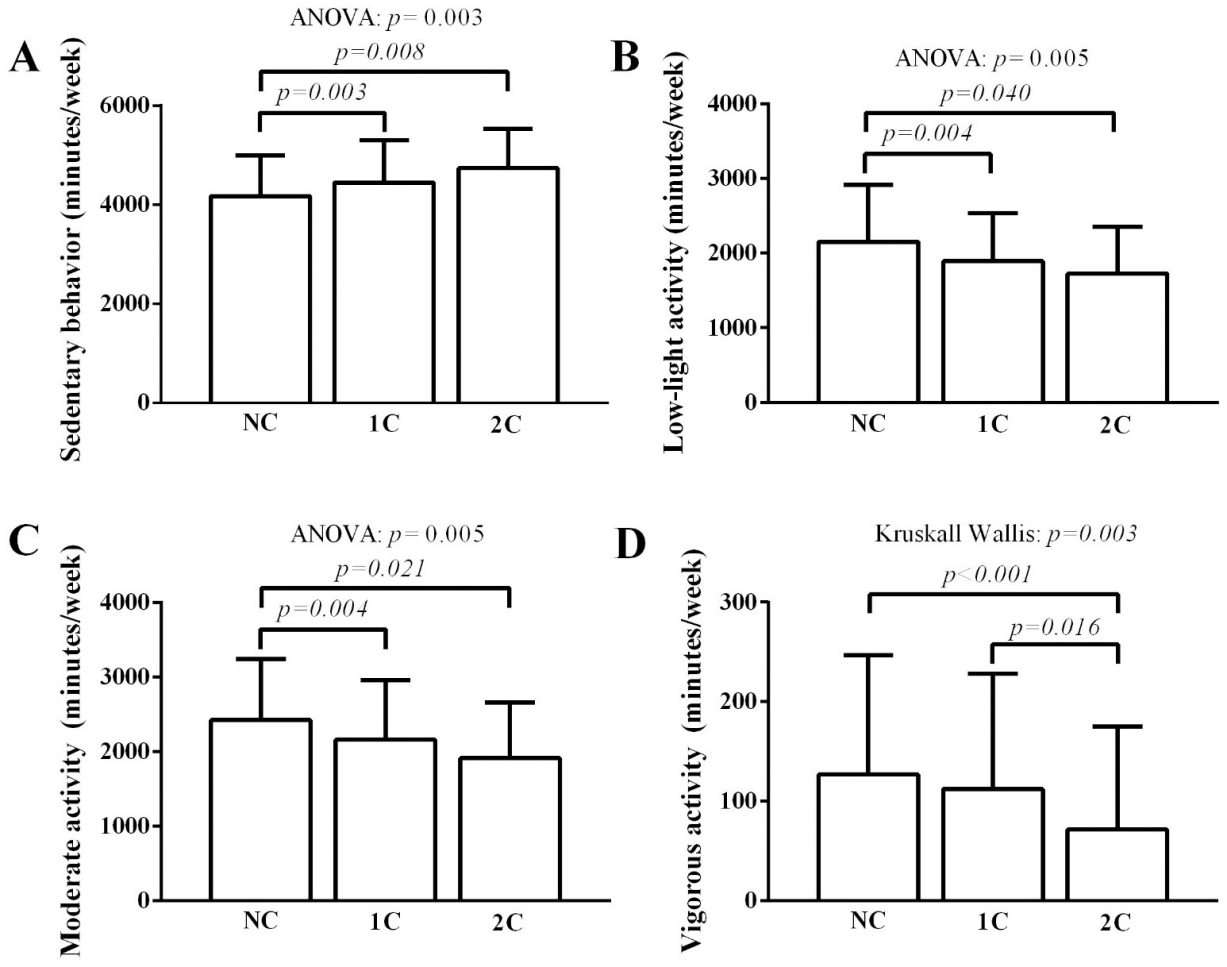
Comparison of the movement behavior outcomes among groups.

Fig 3 shows the comparison of the movement behavior outcomes among the groups. Patients from the 1C and 2C groups spent more sedentary time compared to patients from the NC group (NC: 3948 ± 846 minutes/week vs. 1C: 4539 ± 810 minutes/week vs. 2C: 4564 ± 807 minutes/week, p = .003) (panel A). The patients from the NC group spent more time in low-light physical activity than the patients from the 1C and 2C group (NC: 2291 ± 680 minutes/week vs. 1C: 1826 ± 649 minutes/week vs. 2C: 1885 ± 651 minutes/week, p = .005) (panel B). Patients from the NC group spent more time in moderate physical activity (NC: 2617 ± 796 minutes/week vs. 1C: 2071 ± 767 minutes/week vs. 2C: 2092 ± 776 minutes/week, p = .005) (Panel C). The patients from the NC group spent more time in vigorous physical activity than the patients from the 2C group (NC: 155 ± 148 minutes/week vs. 1C: 110 ± 110 minutes/week vs. 2C: 64 ± 70 minutes/week, p = .003) (Panel D).

Multiple linear regression indicated that the number of fitness components was associated with low-light, moderate, and vigorous physical activity levels (β = -182 [78] minutes/week, p = .021; β = -234 [93] minutes/week, p = .012; β = -38 [12] minutes/week, p = .002), and sedentary time (β = 273 [97] minutes/week, p = .006) independently sex, age and ABI.

The crude and adjusted association between the joint profiles of cardiorespiratory fitness and muscle strength with the movement behavior outcomes are presented in Table 2. The crude analysis indicated a negative and significant association between the joint profiles of cardiorespiratory fitness and muscle strength and low-light and moderate physical activity levels (β = -178 [82] minutes/week, p = .031; β = -236 [96] minutes/week, p = .016) and a positive and significant association with sedentary time (β = 281 [219] minutes/week, p = .006), although this relationship became non-significant after adjustment for covariates.

**Table 2:** Crude and adjusted association between the joint profiles of cardiorespiratory fitness and muscle strength with the physical activity level and sedentary behavior in patients with symptomatic peripheral artery disease.

Outcomes	Exposure	Models	β (SE)	b	P	P_model_
Vigorous activity (min/w)	Joint profiles (NC, 1C and 2C)	Crude	-46 (13)	-.265	.001	< .001
		Model 1	-38 (12)	-.223	.002	< .001
		Model 2	-36 (12)	-.208	.004	< .001
		Model 3	-30 (13)	-.172	.025	< .001
Moderate activity (min/w)	Joint profiles (NC, 1C and 2C)	Crude	-236 (96)	-.194	.016	.016
		Model 1	-234 (93)	-.193	.012	< .001
		Model 2	-170 (92)	-.139	.067	< .001
		Model 3	-100 (95)	-.083	.295	<0.001
Low-light activity (min/w)	Joint profiles (NC, 1C and 2C)	Crude	-178 (82)	-.173	.031	.031
		Model 1	-182 (78)	-.177	.021	< .001
		Model 2	-131 (78)	-.127	.094	< .001
		Model 3	-74 (81)	-.072	.362	< .001
Sedentary behavior (min/w)	Joint profiles (NC, 1C and 2C)	Crude	281 (101)	.219	.006	.006
		Model 1	273 (97)	.212	.006	< .001
		Model 2	206 (96)	.160	.034	< .001
		Model 3	130 (99)	.103	.191	< .001
Steps per week	Joint profiles (NC, 1C and 2C)	Crude	-7874 (1766)	-.339	< .001	< .001
		Model 1	-7346 (1715)	-.316	< .001	< .001
		Model 2	-6247 (1723)	-.273	< .001	< .001
		Model 3	-5014 (1772)	-.228	.005	< .001

β (SE): coefficients regression (standard-error), b: standardized coefficients regression. Joint profiles—NC: those with high strength and cardiorespiratory fitness; 1C: those with at least one component classified as low; 2C: those with both components classified as low fitness. Model 1: adjusted for sex, age and ankle brachial index; Model 2: Adjusted for model 1 plus hypertension, diabetes and dyslipidemia; Model 3: adjusted for model 2 plus coronary artery disease and heart failure.

The crude analysis indicated a negative and significant association between the joint profiles of cardiorespiratory fitness and muscle strength and vigorous physical activity levels (β = -46 [13] minutes/week, p = .001) and step count (β = -7874 [1766] number/week, p = < .001) that remained after adjustment for sex, age and ABI (model 1) (β = -38 [12] minutes/week, p = .002; β = -7346 [1715] number/week, p = < .001), for model 1 plus hypertension, diabetes and dyslipidemia (model 2) (β = -36 [12] minutes/week, p = .004; β = -6247 [1723] number/week, p = < .001) and for model 2 plus coronary artery disease and heart failure (model 3) (β = -30 [13] minutes/week, p = .025; β = -5014 [1772] number/week, p = .005).

## Discussion

The main findings of this study were that PAD patients with low cardiorespiratory and/or strength exhibited an unhealthier movement behavior pattern compared to their physically fit peers. Having at least one low fitness component was associated with reduced step count, low-light/moderate physical activity, and increased sedentary time. However, patients with low fitness in both components are more likely to spend less time engaging in vigorous physical activity.

Impairments in cardiorespiratory fitness have been well described in PAD patients. In this study we employed the total walk distance obtained in the six-minute walk test as a marker of cardiorespiratory fitness. As expected, the results revealed that on average patients with PAD presented walk 30% less than heath matched controls [23]. In same way, low neuromuscular fitness is frequently observed in PAD patients [24, 25].

Contrary to our initial prediction, we found that patients with reduced cardiorespiratory and strength did not have lower low-light/moderate physical activity levels, step count, and increased sedentary time than those with only one component impacted. However, low fitness in both components is related to less time spent in vigorous physical activity. This implies that deterioration in either cardiorespiratory fitness or strength is enough to alter the most indicators related to physical activity levels of PAD people, except for the time spent in more vigorous physical activity, which is impacted only when both components are deteriorated. Although the mechanisms underlying this response were not investigated in our study, previous research has found a link between poor cardiorespiratory or strength and a variety of variables that may contribute to decreased physical activity levels in people with PAD [5–7, 18, 26]. These factors include an earlier onset of pain [27], greater fatigue [18], stair climbing difficulty [28], and poor psychological well-being [29]. It is conceivable that diminished cardiorespiratory fitness and muscle strength could negatively impact low-light/moderate physical activity levels due to common underlying mechanisms, rather than interactive ones.

The absence of interaction between cardiorespiratory fitness and strength concerning low-light/moderate physical activity levels implies that PAD individuals could potentially gain from interventions aimed at enhancing either of these aspects. It is important to highlight that most of these patients’ daily activities are performed at low-light/moderate intensities. However, worth noting is the dearth of studies examining the impact of walking or resistance exercise therapy on the daily physical activity levels of these patients. Subsequent clinical inquiries could address this gap to ascertain whether enhancements in cardiorespiratory fitness and/or strength might elevate daily physical activity among this patient group.

There are limitations of this study that should be addressed. Firstly, the cross-sectional design limits the establishment of causality and temporal correlations. Second, strength and cardiorespiratory fitness were measured using functional tests, which might be influenced by other aspects of physical fitness such as balance, flexibility, and agility. Therefore, the results may not solely reflect the participants’ specific cardiorespiratory and strength levels. Furthermore, the cutoff points for cardiorespiratory fitness and strength were determined using statistical analysis, which may add some subjectivity and fluctuation depending on the sample. Third, although, the 6-minute walk test and the sit to stand test have been used to estimate cardiorespiratory capacity and lower limb strength, respectively, they have also been used to assess the efficacy of different therapeutics interventions [30–32], as well as mortality predictors [33, 34]. They are easy and inexpensive tools, which increases the practical applicability of the current study. Fourth, we did not assess the past history of participants physical activity level. Finally, in this research project blood data was not assessed because they provide limited information in addition to clinical data. As observed in Table 1, our sample present several comorbid conditions and are using several types of medication, factors that directly affect blood biomarkers.

## Conclusions

PAD patients with low cardiorespiratory and/or neuromuscular fitness exhibit an unhealthier movement behavior pattern compared to their physically fit peers, such as a lower step count, reduced levels of low-light and moderate physical activities, and a higher amount of sedentary time. Furthermore, patients with low fitness in both components are more likely to spend less time engaging in vigorous physical activity.

## Supporting information

S1 Data (XLSX)
